# *Ex vivo* generation of umbilical cord blood T regulatory cells expressing the homing markers CD62L and cutaneous lymphocyte antigen

**DOI:** 10.18632/oncotarget.26097

**Published:** 2018-09-14

**Authors:** Joshua N. Kellner, Eric Yvon, Simrit Parmar

**Affiliations:** ^1^ Department of Experimental Therapeutics, The University of Texas MD Anderson Cancer Center, Houston, TX, USA; ^2^ Department of Lymphoma and Myeloma, The University of Texas MD Anderson Cancer Center, Houston, TX, USA; ^3^ Department of Medicine, Hematology and Oncology, George Washington University School of Medicine, Washington, D.C, USA

**Keywords:** regulatory T cells, immunotherapy, cell therapy, ex vivo expansion, cord blood

## Abstract

Regulatory T cells (Tregs) are an important component of the immune system involved in regulation of immune cell proliferation and inflammatory responses and preventing autoimmune diseases. The use of Tregs in cellular therapy has recently been explored in clinical trials specifically evaluating the role of *ex vivo* expanded Tregs in the prevention of graft-versus-host disease during stem cell transplantation. The possibility of Treg use in the clinic requires clinical grade expansion of Tregs for development of cell therapy protocols and proper homing of Tregs to the intended target. Here we demonstrate a novel medium composition to expand CB Tregs, specifically upregulation the homing and activation markers CD62L and cutaneous lymphocyte antigen (CLA). CLA expression was uniquely acquired during activation of Tregs with subsequent loss or lack of expression with media change. This finding highlights the importance of proper growth conditions unique to Tregs that can alter expression of proteins and establishes a baseline for expanding marker specific Tregs that home and target unique tissues.

## INTRODUCTION

Regulatory T cells (Tregs) are a naturally occurring subset of T cells that regulate many processes including immune homeostasis, prevention of inflammation during pathogenic exposure and inhibiting autoimmune development. In all disease states, uncontrolled conventional T cells (Tcon) proliferation leads to the primary mechanisms of subsequent disease. While pharmacological agents have been used to induce anergy of conventional T cells, excessive toxicities or lack of effective targeting has led investigators to pursue other therapies. Immunotherapy strategies have been widely used to regulate the immune system by either preventing proliferation or enhancing immune signals that may be inactivated in the patient. While the use of Tregs for immunotherapy had been previously suggested, technical aspects regarding proper source, isolation and expansion have limited practicality. Initial identification and subsequent expansion studies were done with peripheral blood (PB) Tregs due to convenient access [1, 2]. However, the safety and access to umbilical cord blood (CB) has provided an alternative source for Tregs and while success in expansion [3] and clinical efficacy [4, 5] has been reported, CB Treg function and phenotype is not well defined. In a recent comparative study, PB Tregs were limited in suppressive function when compared to CB Tregs [6]. Since isolation numbers and thus expansion is dramatically lower using CB, improving and optimizing expansion protocols of CB Tregs could overcome the difference in total expansion number achieved with *ex vivo* culture of PB Tregs.

Improved Treg isolation techniques from various sources has significantly improved through the use of automated systems, ultimately improving total yield, expansion, purity and numbers [1, 7–9]. *Ex vivo* expansions have typically involved the addition of IL-2, at various concentrations, and either CD3/28 beads or antigen presenting cells as means of inducing Treg activation [1–3, 7]. These cultures were initiated in either XVIVO15 (XVIVO) (Lonza), a serum-free medium marketed for hematopoietic cells but optimized for proliferation of tumor infiltrating cells (TIL), or RPMI1640, widely used for culturing various cell types. While *ex vivo* cell expansion with each of the above mentioned medium has been successful, none were specifically optimized for Treg cultures and currently it has not been established whether certain media can provide improved or different Treg populations for cellular therapy.

The impact from deficient *ex vivo* expansion of Tregs was suggested due to lack of efficiency in early clinical trials [10]. The L-selectin (CD62L) and the Cutaneous Lymphocyte Antigen (CLA) are surface markers that provide homing and localization signals specific to tissues and were lacking in early *ex vivo* Treg expansions [11]. In fact, fucosylation of *ex vivo* expanded CB Tregs, enforcing CLA expression on the surface, could prevent graft-versus-host disease (GVHD) and promote longer survival in a xenogenic murine model due to more efficient homing [12]. This was further established in clinical settings where *ex vivo* fucosylation of hematopoietic stem cell transplants promoted faster engraftment in patients, attributed to the accelerated homing from the increased CLA expression [13]. Better understanding and development of *ex vivo* expansion cultures specific to Treg proliferation and targeting unique marker subsets of Tregs could promote further studies of Tregs into various other cell therapy protocols.

Here we demonstrate an alternative method of expanding and characterizing CB Tregs using two separate cell culture mediums, XVIVO-15 and SCGM. Overall, Treg phenotype and suppression were largely unimpacted with similar expression levels and suppressive function demonstrated in both medium. However, differences in homing markers were detected, specifically with significant expression of CLA on expanded CB Tregs. In a xenogenic model of GVHD, we discovered that SCGM expanded Tregs were comparable in preventing onset and progression of GVHD. Further, Tregs cultured in SCGM was required for natural expression of CLA on the surface of Tregs, with expression correlating with the Treg activation status.

## RESULTS

**Figure 1:**
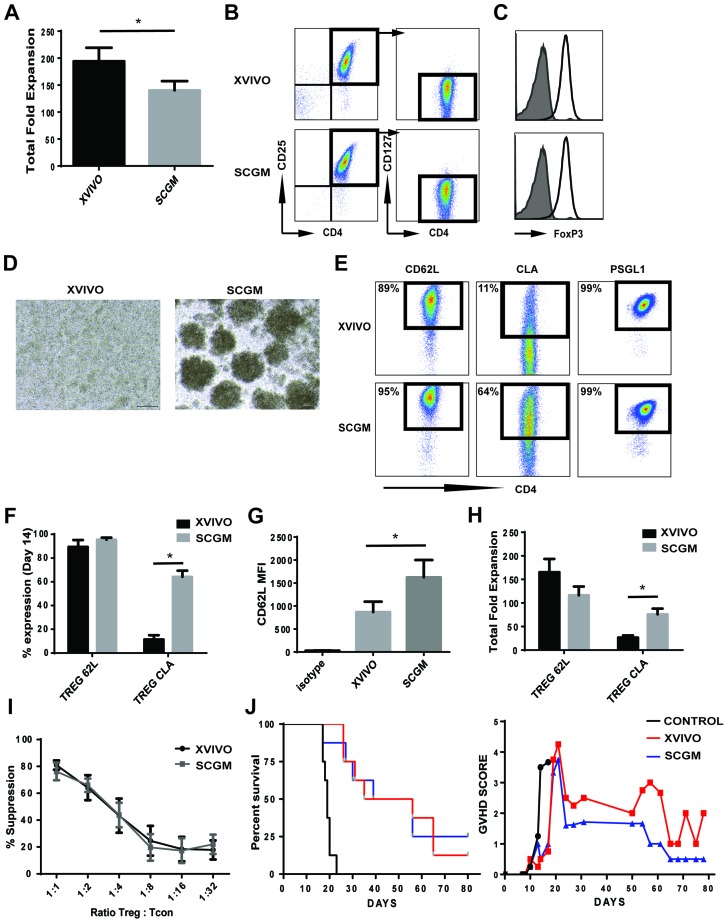
*Ex vivo* expansion of CB Tregs in XVIVO and SCGM **(A)** Total nucleated fold expansion of CB Tregs in XVIVO or SCGM. N=8, p<0.05. **(B)** Flow cytometric representation of CB Tregs (CD4+CD25+CD127-) in XVIVO or SCGM. **(C)** FoxP3 expression in CB Tregs grown in XVIVO (top) or SCGM (bottom). **(D)** Microscopic picture of CB Tregs at Day 7 growth in XVIVO or SCGM. **(E)** Representative flow cytometry plots of CB Treg CD62L (Left), CLA (middle) and PSGL1 (right) expression cultured in XVIVO or SCGM. **(F)** Total % expression of CD62L and CLA in CB Tregs. N=4, p<0.05. **(G)** CD62L MFI expression of CB Tregs cultured in XVIVO or SCGM. N=4, p<0.05. **(H)** Total fold expansion of CD62L or CLA expressing Treg cells in XVIVO or SCGM. N=4, p<0.05. **(I)** Total % suppression of Tcons (CD4+) by CB Tregs cultured in XVIVO (dark bar) or SCGM (light bar). N=4. **(J)** Kaplan-Meier survival curve of NSG mice in GVHD xenogenic model (left panel). GVHD scoring of mice (right panel). N=8. In Figure, error bars depict mean ± SEM.

Expansion protocols for Tregs have utilized various media which have provided robust expansion and the ability to generate clinical protocols. XVIVO was initially developed for expansion of lymphokine activating killer cells and tumor infiltrating lymphocytes as well as for monocytes, macrophages and dendritic cells (DC), and not fully optimized for Tregs thus potentially impacting proper development and proliferation of Tregs. We tested whether SCGM, another good manufacturing practice (GMP) medium typically used for Hematopoietic Progenitor Cells (HPC), Natural killer cells (NK) and Cytokine induced killer cells (CIK) expansion, could significantly expand CB Tregs (Figure 1). After 14 days in culture, XVIVO expanded Tregs 194 ± 25 fold while SCGM induced 140 ± 17 fold expansion (Figure 1A). Both cultures maintained a similar Treg phenotype via flow cytometry, CD4+CD25+CD127- (XVIVO: 95.2 ± 2%, SCGM: 96.4 ± 1.5%), with high expression of FoxP3 in both cultures (Figure 1B, 1C).

During early time points in cell culturing (3-5 days), Tregs in SCGM demonstrated proliferation in unique colony growth suggesting potential adhesion/cell-cell interaction in SCGM (Figure 1D). We assessed the expanded Tregs for markers specific for Treg adhesion and homing (Figure 1E-1H). CD62L, an important receptor for Treg adhesion and migration, was similar in % expression between both cultures, 89 ± 6% XVIVO and 95 ± 2% SCGM (Figure 1E, 1F), however Tregs cultured in SCGM had significantly higher mean fluorescence intensity (MFI) compared to XVIVO (1626 ± 374 and 870 ± 225, respectively) (Figure 1G). Cutaneous lymphocyte-associated antigen (CLA), a specialized form of P-selectin glycoprotein ligand 1 (PSGL-1), enables T cell homing to tissue sites via enhanced binding to CD62E and CD62P. Tregs from both medium demonstrated >99% PSGL-1 expression (Figure 1E). However, CB Tregs expanded in SCGM had 64 ± 5 % expression of CLA while XVIVO expressed only 11 ± 3.5 % (Figure 1E, 1F). The increased CLA expression correlated into significantly more CLA+ Tregs expanded in SCGM compared to XVIVO (76 ± 12 fold and 27 ± 5 fold, respectively) (Figure 1H).

To test whether suppression was impacted, we used a CellTrace assay to examine for proliferation of Tcons *in vitro*. We observed similar rates of suppression of Tcons from either medium, suggesting SCGM expanded Tregs are not inferior (Figure 1I). To further confirm functional response, we used a mouse model of GVHD to test for the suppressive function of expanded CB Tregs *in vivo* (Figure 1J). While control group (PBMCs alone) had a median survival of 19 days, both XVIVO Tregs and SCGM Tregs had similar rates of survival (44 or 48 days respectively) (left panel). Though GVHD scoring of mice did not demonstrate any statistical significance between Tregs cultured in XVIVO or SCGM, SCGM CB Tregs maybe slightly more effective (minimized GVHD peaking; Day 21 and overall scoring; Day 24) suggesting better targeted control of inflammation.

**Figure 2:**
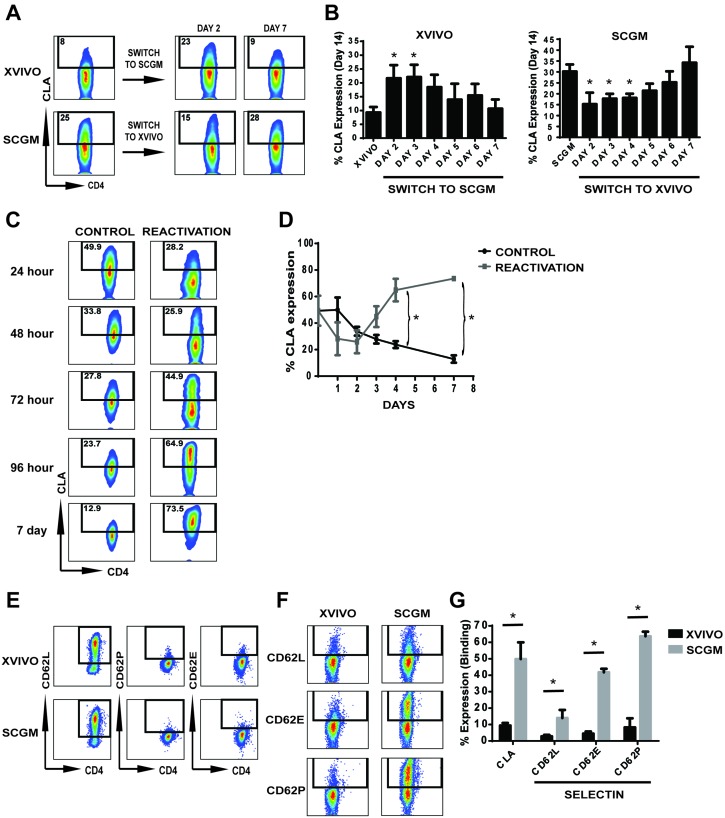
CLA expression and binding on CB Tregs expanded in different mediums **(A/B)** CD25+ cells were intiated in either XVIVO or SCGM and switched to opposite media at Day 2, 3, 4, 5, 6 or 7 and cultured in switched media through 14 days. (A) Representative flow cytometry of CLA expression from Day 14 expanded Tregs in either XVIVO or SCGM (left flow panels) and when switched at Day 2 or 7. (B) Quantitative graph depicting CLA expression on harvest at Day 14. N=3. P<0.05 compared to either XVIVO alone (left panel) or SCGM along (right panel). **(C/D)** SCGM expansion cultures were harvested at Days 10/11 to initiate secondary activation test. Cultures were seeded with or without CD3/28 beads and cultured for an additional seven days. (C) Representative flow cytometric analysis of CB Treg CLA expression after reactivation with CD3/28 beads at each timepoint. (D) Graph depicting CLA expression with or without reactivation. N=5, p<0.05. **(E-G)** Selectin expression and binding analysis of expanded CB Tregs. (E) Expression of CD62L, CD62E and CD62P on the surface of XVIVO or SCGM expanded CB Tregs. (F) Representative flow plot of selectin Fc chimera binding of CD62L, CD62E or CD62P to CB Tregs. (G) Quantitative analysis of Selectin Fc chimera binding to CB Tregs from XVIVO or SCGM. N=4, p<0.05. Throughout Figure, error bars depict mean ± SEM.

Since CLA expression on XVIVO expanded CB Tregs was minimal while present on SCGM expanded CB Tregs, we initiated an experiment to test whether cell culture medium can directly impact CLA expression (Figure 2A, 2B). CB CD25+ cells were initially seeded in either XVIVO or SCGM and individual cultures were removed selectively at Days 2 through Day 7 and placed into opposing media (XVIVO to SCGM; SCGM to XVIVO). Cultures were then maintained in the switched media through 14 days. Figure 1A shows representative flow of CLA expression on cultures at Day 14 (left flow panels) and from cultures switched at either Day 2 or Day 7. As seen in Figure 2B left panel, when XVIVO cultured CB Tregs were switched early into SCGM medium (Days 2/3), CLA expression levels were significantly higher compared to XVIVO (14 days) similar in expression to SCGM (14 days) (Figure 2B, right panel). SCGM cultures switched early into XVIVO medium at Days 2/3 prevented CLA expression on expanding CB Tregs (Figure 2B, right panel). SCGM cultures, switched at Day 7 into XVIVO, did not impact CLA expression exhibiting similar levels to SCGM only cultures (14 days). This suggests that substrate may be present at higher levels in SCGM medium compared to XVIVO and substrate presence may be required immediately following activation for expression of CLA. To test the role of activation in inducing CLA expression, we removed actively expanding CB Tregs in SCGM (Day 10/11) and initiated an expansion culture with or without secondary CD3/28 bead reactivation (Figure 2C, 2D). Figure 2C represents surface expression of CLA from SCGM culture in secondary cultures after 1 day through 7 days. At initiation (Day 10/11 primary expansion), CLA expression was 49.3 ± 11.3 %, which slowly decreased in expression over 7 additional days of culture (Control, 12.9 ± 2.7 %). Upon reactivation, CLA expression initially drops within the first two days before significantly increasing through 7 days (Reactivation, 73.5 ± 1.8%).

The natural binding receptors of CLA during homing and migration is to selectins, specifically E-selectin and P-selectin. We tested the binding efficiency of Tregs to selectin to determine if any impact is conferred on Tregs from *ex vivo* expansion of CB Tregs in SCGM or XVIVO. Figures 2E-2G involve selectin study. We first tested endogenous expression of selectins on expanded CB Tregs, observing expression of only CD62L while lacking both CD62P and CD62E on the surface (Figure 2D). We then examined the binding capacity of expanded Tregs to selectin Fc chimera (Figure 2F, 2G). Quantitatively, while XVIVO expanded CB Tregs demonstrated only 9.3 ± 1.6% total CLA expression, minimal binding to CD62L, CD62E and CD62P was observed (CD62L 2.8 ± 0.9%, CD62E 4.4 ± 1.3% and CD62P 8.3 ± 5.5%). However, CB Tregs expanded in SCGM demonstrated 50 ± 10.2% CLA expression which corresponded to significant binding to CD62L (14 ± 4.9%), CD62E (41.8 ± 2.1%) and CD62P (63.7 ± 2.8%) compared to XVIVO. This further demonstrates that SCGM medium is a potent expander of CLA+ CB Tregs possessing significant selectin binding properties.

## DISCUSSION

Cell therapy protocols have become important in the development of novel clinical approaches in treating patients of various diseases. The low frequency of Tregs in CB MNCs (0.5-2 %) require optimal expansion to achieve clinically relevant cell numbers for therapy. Advancement of cell culture expansions is vital to developing a cell product tailored specifically for treating specific diseases. While Tregs have been shown to be involved in promoting disease, uncontrolled T cell proliferation and subsequent inflammation contribute to many different diseases, of which Tregs could be useful in cellular therapy treatments. Development of Treg expansion cultures and better understanding of proliferating Tregs specific to that expansion could enable generation of optimal cell therapies tailored to specific diseases.

XVIVO and RPMI1640 have been most frequently utilized in *ex vivo* expansion cultures of Tregs, whether from CB or PB sources [7]. However, neither were specifically formulated for optimal expansion of Tregs. Since clinical or industrial manufacturing of Tregs for cellular therapy requires uninterrupted access to components needed for expansion, our primary goal was to compare the differences between two GMP grade mediums, XVIVO and SCGM, to determine if medium could be interchangeably used for CB Treg expansions. While no difference in phenotype was observed and total cell expansion was only slightly impacted, alterations in homing marker expression of the cells was significantly increased in SCGM medium. CD62L, commonly found at high levels in both murine and human Tregs [14], is critically important for Treg function and essential to Treg homing to lymph nodes [15]. It is unclear whether the higher expression of CD62L present on the SCGM expanded Tregs can have differing function compared to XVIVO.

However, most unique was the dramatic increase of CLA present on the surface of SCGM expanded Tregs. Our studies demonstrated minimal expression of CLA on the surface of freshly isolated CB CD25+ (~3%) (data not shown) suggesting that the expression in culture is mechanistic due to activation and availability of substrate (GDP-fucose). Conversely, expression of CLA on PB CD25+ cells was much higher (~55-65%) (data not shown) than CB suggesting either CB and PB Tregs are different in function or they are in different states of activation from their respective tissues. Differences in expression of CLA on CB and PB Tregs has recently been reported [16]. Lack of CLA expression on infused clinical products has been suggested as the reason for lack of efficacy in Treg clinical trials [10, 17]. A recent clinical trial was initiated to test whether enforced fucosylation on CB Tregs infused prophylactically would improve homing and prevent GVHD in HSCT transplantation settings (Kellner et al. unpublished findings). Recent evidence has confirmed that Tregs express fucosyltransferases, important enzymes in establishing fucose on siayl-Lewis x thus creating the CLA determinant on PSGL-1 [18]. The significant increase in CLA induced by the *ex vivo* culturing of CB Tregs in SCGM medium could supplant the need for exofucosylation of Tregs upon harvesting the expansion culture. Further since fucosylation was natural and not enforced, it may not contribute to ancillary effects such as the fever exhibited in patients infused with exofucosylated Tregs (Kellner et al. unpublished findings).

While not statistically significant, suppression assays both *in vitro* and *in vivo* seemed to suggest better efficacy with Tregs cultured in SCGM compared to XVIVO. Though SCGM Tregs demonstrated CLA expression with higher levels of CD62L, our *in vivo* model might not have enough sensitivity to determine whether this could have biological impact such as providing improved adhesion and homing compared to XVIVO. When mice were sacrificed at end of experiment (Day 110), there were significantly more T cells remaining in the XVIVO transplanted mice than the SCGM transplanted mice (data not shown). This suggests that SCGM Tregs established faster suppression of T cells post-transplantation, which is suggested by the lower overall GVHD scoring. Future work involving *in vivo* imaging and immunohistochemistry would be required to effectively determine whether homing advantages exist with expansion of Tregs in SCGM, though limitations specific to the GVHD xenogenic model may exist.

It is unclear what component is present in SCGM that is either null or limited in XVIVO that induces CLA expression, though GDP-fucose may be part of the mechanism. However, two important discoveries emerge from this study into media formulations and gene expression (i.e. CLA); one: substrate is needed early (within 3 days) to allow for effective fucosylation of PSGL1 and two: enzyme (fucosyltransferases) is upregulated during activation of Tregs that enables the process of fucosylation of PSGL1. Further questions arise regarding whether other cells types, T cells or NK cells, have potentially important functional mechanisms occurring immediately after activation and induction of proliferation that may impact cell expansion.

This study also demonstrates the importance of finding an effective culture (medium and components) developed specifically for the target cell to be expanded. The use of SCGM provides both robust expansion, as obtained with XVIVO, while promoting CLA expression, which does not require further manipulation of cells with enforced fucosylation. More specifically, Treg *ex vivo* expansion in SCGM could be used when CLA+ Tregs are needed in cell therapy to home to specific tissues such as with prevention of acute GVHD in skin or gut or minimizing inflammatory skin insults incurred from UV irradiation, allergic dermatitis or from psoriasis [19, 20]. The possibility of targeting specific tissues with Tregs by inducing marker expression during *ex vivo* expansion has been established providing some support to this notion [21]. While we observed little differences in expression of ICOS, LAG3, CTLA4, TIM3 or PD1 between Tregs expanded in XVIVO or SCGM (data not shown), other markers may express differently that could justify their specific use in expanding a certain Treg subtype. We did not attempt to determine if expansion in either medium could dramatically improve total cell numbers after 14 days culture, which would require second reactivation of Tregs. However, this leads to discussions regarding whether Tregs cultured for 7, 14, 21 or 28 days or even after multiple reactivations are similar in phenotype and function and whether such expansions may be futile and potentially deleterious to the reason for expanding Tregs. Hippen *et al.* showed that though additional signaling via 4-1BB improved total Treg expansion from CD32 stimulation alone, some loss of suppression is incurred [7]. Optimizing culture media to complement the unique signaling that promotes significant *ex vivo* expansion while maintaining potent Treg suppressing populations is key to developing a Treg *ex vivo* expansion protocol for specific cellular therapy.

## MATERIALS AND METHODS

### Cord blood

CB products were obtained under MD Anderson Cancer Center Institutional Review Board-approved protocols with informed consent. Blood was layered over Lymphoprep (StemCell Technologies, Vancouver, BC, Canada) and mononuclear cells (MNCs) were collected from buffy coat.

### Animal studies

Non-obese diabetic/SCID/IL2Rγ null (NSG) mice were obtained from either Jackson Laboratories (Bar Harbor, Maine, USA) or through breeding colony established at The University of Texas MD Anderson Cancer Center under the Department of Experimental Radiation Oncology. Mice were maintained according to the guidelines and an approved protocol by The University of Texas MD Anderson Cancer Center Institutional Animal Care and Use Committee.

### Cell isolation

#### Selection

CB Tregs were isolated from MNCs using CD25 microbeads (Miltenyi Biotec, Bergish Gladbach, Germany) following manufacturer's instructions.

### Cell culture

CB CD25+ cells were co-cultured with CD3/28 co-expressing Dynabeads® (ClinExVivo™ CD3/CD28, Invitrogen Dynal AS, Oslo, Norway) in a 1:1 cell:bead ratio and seeded at 1×10^6^ cells/ml. Cells were cultured in either XVIVO15 (XVIVO) (Lonza Biowhittaker, Morristown, NJ. USA) or SCGM (Cellgenix, Portsmouth, NH. USA) mediums supplemented with 10% fetal bovine serum (FBS, Sigma, St. Louis, MO. USA), 2 mM L-glutamine (Sigma, St. Louis, MO), 1% Penicillin-Streptomycin (Gibco/Invitrogen, Grand Island, NY)] and 1000 IU/ml interleukin (IL)-2 (CHIRON Corporation, Emeryville, CA). Cells were placed into 37°C incubator with 5% CO_2_-in-air atmosphere. Culture was supplemented with fresh medium and IL-2 every 2-3 days and maintained at 1×10^6^ cells/ml during the time of expansion. Cultures were harvested after 14 days.

### Flow cytometry

CD4, CD8, CD25, CD127, CD62L, CD62E, CD62P and FoxP3 antibodies were obtained from eBioscience (ThermoFisher Scientific, Waltham, MA. USA). CLA antibody was obtained from BD Biosciences (San Jose, CA. USA).

### XVIVO15/SCGM medium switch

CD25+ cells were cultured in either XVIVO or SCGM medium with 1:1 CD3/28 beads and 1000 U/ml IL-2. A fraction of cells was removed from culture and switched into opposite medium at Day 2, 3, 4, 5, 6 and 7 and cultured for 14 days with fresh media added every other day. Once media was switched, cultures remained in the assigned medium. Control cultures were cells grown in either XVIVO or SCGM for 14 continuous days.

### SCGM/activation assay

CB 25+ cells were cultured for 10 days in SCGM, 1:1 beads and 1000 U/ml IL2. At Day 10 or 11, cells were removed and placed into culture with or without beads. Wells were harvested at 24 hr, 48 hr, 72 hr, 96 hr and 7 days to evaluate CLA expression.

### Selectin binding assay

Recombinant Human P, E or L Fc chimera proteins were obtained from R&D Systems (Minneapolis, MN. USA). Anti-human IgG1 secondary antibody was obtained from BD Biosciences. Cells were stained with 0.4 ug of respective Fc chimera protein for 30 minutes at 4°C, washed and stained with secondary antibody for 30 minutes at 4°C. Cells were acquired with FACSCalibur (BD Biosciences) and analyzed with FlowJo software (FlowJo, Ashland, OR. USA).

### Suppression assay

Tcons (CD4+CD25-) were thawed and stained with CellTrace Violet (ThermoFisher Scientific) following manufacturer's recommendation. Tregs and Tcons were placed into 96-well plate at 1:1, 1:2, 1:4, 1:8, 1:16 and 1:32 (Treg:Tcon) ratios. Cells were activated 1:1 with CD3/28 beads. Culture was harvested after 3 days and acquired using LSR Fortessa (BD Biosciences).

### *In vivo* transplantation

NSG mice were sub-lethally irradiated with 300 cGy irradiation. Mice were transplanted intravenously with Tregs (Day-1) or PBMCs (Day 0) at a 1:1 ratio (Tregs 10 × 10^6^: PBMCs 10 × 10^6^). Mice were examined twice weekly for weight loss, GVHD and survival. GVHD scoring was performed using protocol established by Ferrera et al [22]. Survival was determined via Kaplan-Meier.

### Statistics

Data were analyzed for statistical significance using the Student's t test. Data and statistics were performed using GraphPad Prism 6 software (GraphPad, La Jolla, CA. USA).
